# Migraine Prevention through Ketogenic Diet: More than Body Mass Composition Changes

**DOI:** 10.3390/jcm11174946

**Published:** 2022-08-23

**Authors:** Mariarosaria Valente, Riccardo Garbo, Francesca Filippi, Alice Antonutti, Veronica Ceccarini, Yan Tereshko, Cherubino Di Lorenzo, Gian Luigi Gigli

**Affiliations:** 1 Clinical Neurology Unit, Santa Maria della Misericordia University Hospital, 33100 Udine, Italy; 2 Department of Medical Area, University of Udine, 33100 Udine, Italy; 3 Neurology Unit, Hospital of Gorizia, 34170 Gorizia, Italy; 4 Department of Clinical and Experimental Medicine, University of Florence, 50121 Florence, Italy; 5 Department of Medico-Surgical Sciences and Biotechnologies, Sapienza University of Rome Polo Pontino, 04100 Latina, Italy

**Keywords:** ketogenic diet, ketosis, ketones, migraine, headache

## Abstract

The ketogenic diet (KD) is gaining attention as a preventive treatment for migraine, which is sustained by many pre-clinical and clinical data. KD is also used for weight loss, and there is a relation between migraine and weight excess, but it is speculated that KD efficacy on migraine may go beyond this effect. We conducted a retrospective observational study on 23 migraine patients who received a KD and were evaluated at the baseline and then after 3 months both from a neurological and a nutritional point of view, including body mass composition analysis. We observed a reduction in monthly headache days (12.5 ± 9.5 vs. 6.7 ± 8.6; *p* < 0.001) and in days of acute medication intake (11.06 ± 9.37 vs. 4.93 ± 7.99; *p* = 0.008). We also observed a reduction in patients’ weight (73.8 ± 15.2 vs. 68.4 ± 14.6; *p* < 0.001) and BMI (26.9 ± 6.2 vs. 23.7 ± 8.1; *p* < 0.001) with a decrement of the fat mass (28.6 ± 12.5 vs. 20.6 ± 9.8; *p* < 0.001). Patients who responded to KD and those who did not had no differences with respect to weight or fat mass loss. These data corroborate the utilization of KD as a preventive treatment for migraine and suggest that the efficacy of such an intervention is not only due to weight or fat mass loss but probably relies on other mechanisms specific to KD.

## 1. Introduction

Migraine is one of the most common neurological disorders, with a prevalence of about 12% of the general population [1], and it is also a highly disabling condition which has been estimated to cause 45.1 million years of life lived with disability in 2016 [2]. Despite the great amount of possible choices for prophylactic treatment, classical oral preventive anti-migraine treatments are effective in only 40–50% of the patients, and they are also on the whole poorly tolerated [3]. Monoclonal antibodies targeting calcitonin gene-related peptide (CGRP) have proved great efficacy in migraine treatment and brought about a revolution in headache centers. However, these drugs are not considered a first-line treatment by now, are subjected to prescribing limitations, and consequently are not available for all migraine patients. In addition, despite their great safety [3], some contraindications are present, and, as for all other drugs, there are patients who may not respond to the treatment.

Given these considerations, there is a lot of interest and research focusing on non-pharmacological options for the treatment of migraine, which include nutraceuticals, psychological therapies, behavioral techniques such as relaxation strategies and biofeedback therapy, physical therapy, acupuncture, exercise and neuromodulation [4,5]. Since evidence suggests a relation between diet and headache [6], and furthermore weight loss can be effective in improving migraine [7], different nutritional interventions have also been studied as preventive treatments for this disorder [8].

The ketogenic diet (KD) is a regimen that mimics fasting and induces ketone bodies production. This is obtained through carbohydrate restriction, with the aim of decreasing insulin secretion and increasing glucagon secretion along with the mobilization of fatty acids from adipose tissue to the liver for their β-oxidation and production of ketone bodies [9]. In practice, ketone bodies are formed from ingested fatty acids while using ketogenic high-fat diets, or from endogenous fatty acids, as in hypoglucidic–hypolipidic–normoprotein diets such as very low-calorie ketogenic diets (VLCKDs) [10]. Different types of KD are also distinguished on the basis of the ketogenic ratio, which is the ratio between the weight of fat versus that of protein and carbohydrates contained in the diet. Diets with a lower ketogenic ratio determine lower blood ketones levels but more easily meet protein requirements, are less restrictive, and are more palatable [10]. KD has been initially developed for epilepsy treatment in children almost a century ago following the observation that this condition could ameliorate with starvation [11], and it is now also used in adults with drug-resistant epilepsy [12]. It has recently gained attention in the field of headache after the description of some cases of migraine improvement with such a dietetic regimen [13,14]. After these initials observations, evidence supporting an efficacy of KD in migraine treatment rapidly grew [15,16,17], and clinical recommendations on optimal practice to treat patients with headaches using KD have recently been published [10].

Migraine has been related to weight and adiposity excess [18], and low-grade inflammation and hormonal changes may be the major actors of this relationship [19]. Consequently, weight loss proved to be effective in migraine reduction [7]. KD, although developed in a neurological setting [11], is currently used also for weight loss [20] and can effectively reduce fat mass [21,22]. Given these considerations, this study aims at clarifying if the observed effect of KD on migraine is due only to weight and fat mass reduction or not, corroborating in this second case a specific action of KD on migraine. 

## 2. Materials and Methods

### 2.1. Participants

We performed a retrospective observational study including all patients diagnosed with migraine according to The International Classification of Headache Disorders 3rd edition [23] who were referred by our headache center to our nutritional service from January 2020 until the end of June 2021 to receive a KD for therapeutic purposes and who accepted to start the diet. Only the patients who gave written informed consent for their data utilization for research purposes were considered in the study. 

### 2.2. Procedure

At our institute, migraine patients who are addressed to a KD routinely undergo a basal neurological and nutritional evaluation. During neurological evaluation, clinical data were collected, including comorbidities, the history of current and previous migraine preventive treatments, headache days and days of acute medication intake in the previous month. During nutritional evaluations, patients consistently underwent weight determination, bioimpedance analysis using BIA 101 BIVA PRO (Akern^®^) and food preferences collection. Based on these data, a KD was prescribed specifically tailored to the patients’ characteristics, and they were educated to properly follow the diet. After three months, patients were re-assessed both from a neurological and a nutritional point of view to evaluate diet efficacy, tolerability, compliance and patients’ global impression of change (PGIC) [24]. Clinical and nutritional data at the baseline and at three months follow-up visits were acquired by means of medical records. Patients presenting at least 50% headache frequency reduction were considered responders to the treatment. 

### 2.3. Statistical Analysis

Statistical analysis was performed using JASP version 0.14.1 for macOS (University of Amsterdam, the Netherlands). Characteristics were compared between different groups using a *t*-test, paired samples *t*-test, Mann–Whitney’s test, Wilcoxon’s test or Fisher’s exact test as appropriate. A Shapiro–Wilk test correction was used to assess the normal distribution of data. Two-tailed *p*-values of <0.05 were considered statistically significant.

## 3. Results

A total of 33 patients satisfying inclusion criteria were included. Of these, four patients discontinued the diet before reaching the three months evaluation: two of them because of poor compliance, one due to reported inefficacy and one due to excessive weight loss. Two other patients were lost at follow-up without known reasons. Of the 27 remaining patients, 23 had undergone the 3-month evaluation at the time of data collection and were considered in the final analysis.

In total, 22 out of 23 patients were female and only one was male. The mean age was 47.22 ± 15.21 years. Among the patients, 8 were diagnosed as having migraine with aura, 10 had chronic migraine and 6 had superimposed medication overuse headache. Before diet initiation, patients reported a mean of 12.5 ± 9.5 headache days with 11.06 ± 9.37 days of acute medication intake in the previous month. Table 1 summarizes the patients’ baseline clinical characteristics: 19 patients were also receiving migraine prophylactic therapy, which are summarized in Table 2, and 5 patients were receiving more than one prophylactic drug. Patients had previously tried a mean of 1.78 ± 2.21 prophylactic treatments.

Among the patients, 5 (21.7%) were obese, and 8 (34.8%) were overweight. Table 3 summarizes the patients’ nutritional status at baseline and after three months. Regarding diet, 4 patients were prescribed a VLCKD, 5 patients underwent a KD with a 2:1 ketogenic ratio, 5 patients underwent a KD with a 1.5:1 ketogenic ratio, 8 patients underwent a KD with a 1:1 ketogenic ratio, and 1 patient was prescribed a KD with a 0.5:1 ketogenic ratio.

Out of all the patients, 14 (60.9%) presented at least one comorbidity: fibromyalgia (4) and arterial hypertension (3) were the most frequent ones.

In regard to diet safety and tolerability, considering the 23 patients comprehended in the final analysis, five patients (21.7%) complained of some side effects, including nausea (one patient), fatigue (three patients), constipation (two patients), and abdominal pain (two patients). The reported side effects were generally mild and transient, and only one patient discontinued the diet due to side effects (excessive weight loss; the patient was not considered in the final analysis because he discontinued the diet before the second evaluation).

In regard to the diet efficacy on headache, we observed a significant reduction in monthly headache days from the baseline to the second evaluation after 3 months of diet initiation (12.5 ± 9.5 vs. 6.7 ± 8.6; *p* < 0.001). In total, 17 patients (73.9%) reported a reduction in the headache days, and 15 patients (65.2%) reported a reduction in headache days of at least 50% and were considered as responders. The days of acute medication intake were also significantly reduced, passing from 11.06 ± 9.37 to 4.93 ± 7.99 (*p* = 0.008). PGIC at three months was 4.8 ± 2.3. The initial presence of a situation of chronic migraine or of medication overuse headache was not related to KD response (*p* = 0.379 and *p* = 0.621, respectively). Similarly, the proportion of responders did not significantly differ between patients receiving a concomitant drug treatment or not (*p* = 1.0).

In light of patients’ nutritional status, there was a significant reduction in patients’ weight (73.8 ± 15.2 vs. 68.4 ± 14.6; *p* < 0.001) and subsequently of body mass index (BMI) (26.9 ± 6.2 vs. 23.7 ± 8.1; *p* < 0.001). Importantly, the weight loss was mainly due to the reduction in the fat mass (28.6 ± 12.5 vs. 20.6 ± 9.8; *p* < 0.001), while the lean mass did not change significantly (47.1 ± 8.1 vs. 47.9 ± 7.3; *p* = 0.802), as well as total body, intracellular and extracellular water. The extent of weight and BMI reduction was similar in patients who had normal BMI (<25 kg/m^2^) or who were overweight or obese at baseline (4.22 ± 2.88 vs. 6.48 ± 3.83; *p* = 0.320 for weight; 4.95 ± 8.30 vs. 2.58 ± 1.60; *p* = 0.884 for BMI). Considering the parameters that showed a clear modification during the diet, it is important to note that patients who responded to KD and those who did not had no differences regarding weight loss (5.6 ± 2.7 vs. 6.2 ± 5.1; *p* = 0.299) or fat mass loss (6.1 ± 2.1 vs. 5.0 ± 4.1; *p* = 0.120). In the same way, we observed no statistically significant difference in the reduction in headache days between patients who were normal-weighted or who were overweight or obese at baseline (9.2 ± 11.5 vs. 3.7 ± 3.2; *p* = 0.545).

## 4. Discussion

We reported a significant reduction in headache days and acute medication intake in patients suffering from migraine who underwent a period of KD of at least three months, and the benefit was obtained irrespective to the weight loss and fat mass loss. Although with a small sample, response to the treatment was also observed in patients with chronic migraine and superimposed medication overuse headache. In addition, the diet was found to be safe, and the observed side effects were in line with the literature [10,12] and were generally mild and transient.

The relation between obesity and migraine has been extensively described, with many studies showing an association between these two conditions’ prevalence, frequency and severity [7,19]. Adipose tissue does not just have a storage function, and metabolic signals deriving from nutrients’ excess and energy overload in adipocytes can trigger the activation of intracellular pathways of inflammation. These pathways, alongside with the modifications in adipokines secretion, can promote a state of persistent low-grade inflammation mediated by the recruitment and activation of immune system cells in the adipose tissue and by the production of many inflammatory mediators [25]. Since an increase in levels of different pro-inflammatory mediators has been described both in obesity and migraine [19], and considering that sterile meningeal neurogenic inflammation is one of the assumed mechanism of migraine pathogenesis [26,27], this inflammatory state may be the link between the two phenomena [19]. Hypothalamic dysfunction due to increased food intake and endocrine modifications, especially in sex hormones, which are processed in part in adipose tissue, has been also advocated as possible shared mechanisms between the two conditions [19]. In a recent meta-analysis, it has been demonstrated that weight loss reduces migraine frequency, severity and disability, without regard to whether it is achieved by behavioral interventions or bariatric surgery [7].

Since migraine improvement after weight loss and fat mass reduction may rely on the correction of the above-mentioned mechanisms, and KD is effective and often used for weight loss purpose [20], it could be thought that the effects observed in our group may be due to weight loss. However, we used bioimpedance analysis to document body mass composition before and during the diet, and we observed that responders and non-responders did not differ in terms of weight or fat mass loss. Similarly, in our study, patients with normal baseline BMI did not show a statistically significant difference in headache days reduction when compared with obese and overweight patients. These observations suggests that the efficacy of the proposed dietary intervention does not rely on weight loss but depends on other mechanisms specific to KD.

KD affects metabolism in very different ways, and consequently, many theories to explain its effect on migraine have been proposed. First of all, KD has anti-inflammatory properties that go beyond the effect of fat mass reduction and involve beta-hydroxybutyrate agonism on hydroxy-carboxylic acid receptor 2 (HCA2) and inhibition of the inflammasome [10,28]. Sterile meningeal inflammation is considered to be one of the pathogenetic mechanisms of migraine, which is probably initiated by the release of peptides from trigeminocervical nerve fibers that consequently activates the local immune system. The persistence of this inflammatory reaction is thought to contribute to the sensitization of meningeal nociceptors and central pain pathways, which are at the basis of migraine pathogenesis [27]. Moreover, KD enhances mitochondrial respiration and activates different anti-oxidative pathways such as that of NRF-1, NRF-2 and ERRα [29], and this may be relevant, since migraineurs exhibit increased oxidative stress [10]. The brain in migraine patients tends to present an energy deficit compared to healthy subjects. KD, providing an alternative and efficient source of fuel, may restore this metabolic imbalance. This is possible since the same weight of beta-hydroxybutyrate or acetoacetate can provide more ATP in respect to glucose [10], increasing the phosphocreatine/creatine ratio into the brain, as shown in an experiment on rats [30]. KD may also compensate the imbalance between excitatory and inhibitory neurotransmission in migraine [26]. This effect may be due to the KD facilitation of glutamate conversion to glutamine, allowing efficient glutamate removal and glutamine conversion to GABA [26]. The modulation of adenosine triphosphate-sensitive potassium channels that are opened by KD metabolites, consequently reducing firing in central neurons, may have a role as well [10]. KD effect on central nervous system excitability was well described in a study on migraine patients who were characterized by a baseline increase in visual and somatosensory evoked potentials amplitude responses during repetitive stimulation (deficit of habituation) and in which dietetic intervention was followed by a significant evoked potential amplitude decrement (habituation) in parallel with clinical improvement [31]. Similarly, it has been reported that one month of KD can normalize at the cortical level the typical interictal deficit of habituation of migraineurs as measured by pain-related evoked potentials, although it does not modify such a habituation deficit in the brainstem as measured by nociceptive blink reflex [32]. Interestingly, excessive cortical excitability reduction has been also described to accompany clinical response in other migraine prevention therapies of well-defined efficacy such as onabotulinum toxin A [33]. Conversely, the persistence of deficit of habituation of blink reflex was also observed in patient treated by topiramate and/or biofeedback [34]. In summary, most of the effective treatments for migraine play a role at the cortical level, not at the brainstem, and this is also the case with KD. It is also speculated that gut microbiota may play a role in migraine pathogenesis, and its regulation may be another possible mechanism of action of KD [10]. All this considered, KD action is probably mediated by multiple factors, comprehending ketone bodies, other KD components and reduction in simple carbohydrates [35].

Considering this effects of KD and following the report of some cases of migraine improvement in patients following this kind of diet [13,14], the possibility of using KD in migraine was firstly corroborated by a proof-of-concept study in overweight migraine patients in which one group received a very low-calorie KD for 1 month followed by 5 months of low-calorie diet with progressive carbohydrate reintroduction, while the other received a standard low-calorie diet for 6 months [12]. In this study, headache days reduction was observed in both groups, but it was markedly greater in the KD group after 1 month, and it was followed by a worsening during the period of transition to a standard low-calorie diet, thus suggesting an efficacy of ketosis on migraine [15]. A subsequent double-blind crossover trial compared a VLCKD and a very low-calorie non-ketogenic diet (VLCnKD). During the VLCKD, patients experienced a significant reduction in migraine days with respect to VLCnKD [16]. More recently, an open-label, single arm clinical trial involving patients with chronic migraine and medication overuse headache who were treated with a KD highlighted a reduction in many headache severity parameters [17]. It has to be accounted that not all studies reported KD to be effective. A very recent study compared KD with an “anti-headache” diet based on trigger food avoidance, promoting the consumption of raw foods rather than heavily processed ones and emphasizing adequate hydration and not skipping meals. This study did not show any significant difference between the two interventions. However, the small sample size may have affected the results [36].

There are also some data that point toward a possibility of using low glycemic index diet (LGIT) in migraine prophylaxis [37,38]. This is not strictly a KD, but it is however based on some carbohydrate restriction and consumption of low glycemic index foods [10]. In a study on patients suffering from migraine without aura in which LGIT was compared with standard pharmacological prophylaxis, the two groups experienced a similar reduction in the number of attacks per month [37].

Beyond KD, a small study investigated patients with episodic migraine who received a one-month medium chain triglycerides (MCTs) supplementation without dietary modification [35]. MCTs metabolization consists in the production of energy or, if taken in excess, in the biosynthesis of ketones [10]. In the study, a reduction in monthly migraine episodes and a reduction in episodes duration and secondary migraine symptoms was reported [35]. However, MCTs supplementation may exert some but certainly not all of the effects of a KD.

Interestingly, considering another type of primary headache, a recent study showed a reduction in headache attacks in patients suffering from cluster headache who were treated with a modified Atkins diet, which is another kind of KD [39].

Our study appears to be in line with the literature, since it corroborates the evidence of an efficacy of KD as a possible preventive treatment in migraine. Its novelty relies in the documentation of migraine improvement independently from weight and fat mass loss. Data on this specific aspect in the literature are scarce and have not been previously supported by bioimpedance analysis. In the aforementioned proof-of-concept study by Di Lorenzo et al., KD patients showed a greater reduction in BMI, but when carbohydrates were reintroduced, a rebound of the headache was observed despite the benefit on weight loss [15]. In another study of the same group, VLCKD was significantly more effective than VLCnKD despite inducing similar weight loss [16]. Similarly, in the only published study regarding cluster headache treatment with KD, since neither obese nor overweight patients were included, findings support an effect of KD which is not exclusively mediated by weight loss [39].

The present study has many limitations. First of all, since it is a collection of real-life data, the majority of the patients were treated not only with KD but also with many different pharmacological therapies. However, we think that this better reflects the population of migraineurs generally treated with KD, since KD is considered a reasonable option for drug-resistant migraine [10]. In addition, we did not observe a different proportion of responders when confronting patients on a prophylactic drug with those not on pharmacological treatment, but given the small number of patients involved, this finding must be considered with caution. Although blood beta-hydroxybutyrate was measured in many of our patients, since we did not systematically collect these data, we cannot describe whether there was a relationship between blood ketone levels and headache frequency or severity. It must also be considered that the study has a small sample size, which limits its strength and makes it difficult to perform subgroups analysis, for example to evaluate if patients treated with a different kind of pharmacologic therapies present different responses to KD. Another limit is provided by the short period of time considered: since migraine is a chronic condition, studies with longer follow-up will be needed to determine if the diet has sustained effect and good adherence and safety over time. The study is also limited by its observational and retrospective nature, by the lack of a control group, and by the possibility of a selection bias, since patients were addressed to the nutritional intervention on a clinical basis and liberally decided whether to follow it or not. Finally, as for the majority of studies on dietary interventions, the study was conducted in an unblinded manner.

## 5. Conclusions

In conclusion, we reported KD to be a possible effective and safe preventive treatment for migraine, together with positive effects on weight and fat mass reduction. To our knowledge, this is the first study to take into account body mass composition modification in migraine patients receiving a KD. Since we observed similar modifications of body composition both among patients non-responding to the diet and in patients with good response to the diet, our study indicates that the improvement of migraine after KD treatment is likely due to factors different from weight loss. These observations support the utility of further studies on larger samples and with a prospective design to deepen the knowledge and evaluate the benefits and drawbacks of this nutritional approach in migraine prophylaxis.

## Figures and Tables

**Table 1:** Patients’ clinical characteristics.

Patient Characteristics	Absolute Frequency	Relative Frequency
Female sex	22	95.6%
Migraine without aura	15	65.2%
Migraine with aura	8	34.8%
Episodic migraine	13	47.8%
Chronic migraine	10	43.4%
Superimposed medication overuse headache	6	26.1%

**Table 2:** Patients’ concomitant migraine prophylactic therapy during KD.

Migraine Prophylactic Therapy	Absolute Frequency
Beta blockers	3
Anti-epileptic drugs	6
Antidepressants	7
Onabotulinum toxin A	4
Monoclonal antibodies	4

**Table 3:** Patients’ nutritional characteristics before and after 3 months of KD.

Nutritional Characteristics	Baseline (Mean ± SD)	3 Months (Mean ± SD)	*p*-Value
Weight (kg)	73.8 ± 15.2	68.4 ± 14.6	<0.001
Body mass index (BMI) (kg/m^2^)	26.9 ± 6.2	23.7 ± 8.1	<0.001
Phase angle	5.0 ± 0.7	5.3 ± 0.5	0.422
Lean mass (kg)	47.1 ± 8.1	47.9 ± 7.3	0.802
Fat mass (kg)	28.6 ± 12.5	20.6 ± 9.8	<0.001
Total body water (L)	34.8 ± 4.9	35.1 ± 5.9	0.627
Intracellular water (L)	17.0 ± 3.2	13.6 ± 3.3	0.333
Extracellular water (L)	17.6 ± 2.5	17.2 ± 2.9	0.930

## Data Availability

The datasets analyzed during the current study are available from the corresponding author on reasonable request.
